# d-Cysteine-Induced Rapid Root Abscission in the Water Fern *Azolla Pinnata*: Implications for the Linkage between d-Amino Acid and Reactive Sulfur Species (RSS) in Plant Environmental Responses

**DOI:** 10.3390/antiox8090411

**Published:** 2019-09-18

**Authors:** Hideo Yamasaki, Masahiro P. Ogura, Katsumi A. Kingjoe, Michael F. Cohen

**Affiliations:** 1 Faculty of Science, University of the Ryukyus, Okinawa 903-0213, Japan; 2 Department of Biology, Sonoma State University, Rohnert Park, CA 94928, USA; cohenm@sonoma.edu

**Keywords:** abscission, *Azolla*, d-amino acid, d-cysteine, H_2_S, polysulfide, stress response

## Abstract

Reactive Oxygen Species (ROS) and Reactive Nitrogen Species (RNS) have been proposed as universal signaling molecules in plant stress responses. There are a growing number of studies suggesting that hydrogen sulfide (H_2_S) or Reactive Sulfur Species (RSS) are also involved in plant abiotic as well as biotic stress responses. However, it is still a matter of debate as to how plants utilize those RSS in their signaling cascades. Here, we demonstrate that d-cysteine is a novel candidate for bridging our gap in understanding. In the genus of the tiny water-floating fern *Azolla*, a rapid root abscission occurs in response to a wide variety of environmental stimuli as well as chemical inducers. We tested five H_2_S chemical donors, Na_2_S, GYY4137, 5a, 8l, and 8o, and found that 5a showed a significant abscission activity. Root abscission also occurred with the polysulfides Na_2_S_2_, Na_2_S_3_, and Na_2_S_4_. Rapid root abscission comparable to other known chemical inducers was observed in the presence of d-cysteine, whereas l-cysteine showed no effect. We suggest that d-cysteine is a physiologically relevant substrate to induce root abscission in the water fern *Azolla*.

## 1. Introduction

Plants sense environmental conditions and transmit the environmental signals to regulate their growth and development. It is a long standing question as to how plants sense and transmit a variety of environmental signals without nervous systems [1]. Stomatal movement is one of the rapid responses of plants, both opening and closure movements finish within an hour [2]. Because of this advantage, the stomatal movement has been a model system to be investigated for many years to answer this question [3].

It has been revealed that hydrogen peroxide (H_2_O_2_) is involved in the stomatal closing response [4]. H_2_O_2_ belongs to Reactive Oxygen Species (ROS) which can be produced in abiotic as well as biotic stress conditions [5]. Later, the Reactive Nitrogen Species (RNS) nitric oxide (NO) was found to induce stomatal movements [6,7]. The potential for cross talk between ROS and RNS has become a subject of debate regarding the signaling mechanism in guard cells [8,9]. Early in this century, Reactive Sulfur Species (RSS) was hypothesized as the third group of redox-active molecular species that may be associated with oxidative stress [1,10,11]. In mammalian studies, there is an increasing number of reports suggesting regulatory functions of H_2_S or RSS in cellular signaling mechanisms [12]. Accordingly, it has recently been reported that H_2_S also influences stomatal movements [13]. To date, however, there seems to be contradictory results for the functions of H_2_S in stomatal movements [14].

Researchers may experience technical difficulties in conducting H_2_S experiments with land plants. In whole plant experiments, H_2_S has been delivered by fumigation of the gas [15], spraying of an NaHS solution [16,17], or inclusion of NaHS into a culture medium [18]. Pharmacological comparative studies may be difficult to conduct with those methods. To overcome such technical problems in plant H_2_S study, we suggest here the application of the water-floating fern *Azolla*, a good model plant to explore the physiological functions of chemical compounds [19,20,21].

Plants of the genus *Azolla* have been used for agriculture in East Asia as green manure [22]. Biological research on *Azolla* has a long history in botany [23], cell biology [24,25], and phytoremediation [26,27]. It has long been known that the roots of *Azolla pinnata* are deciduous [28]. In 1993, Kitoh and co-workers found that nitrite (NO_2_^−^) and volatile organic acids such as acetate or propionate contained in swine waste water cause root shedding in *Azolla filiculoides* [29]. Uheda and Kitoh experimentally reproduced the root shedding with a variety of inhibitors, such as the hemeprotein inhibitors sodium azide (NaN_3_) and sodium cyanide (NaCN), the uncouplers 2,4-dinitrophenol (DNP) and carbonyl cyanide *m*-chlorophenyhydrazone (CCCP), and the F_0_-F_1_ ATPase inhibitor *N′,N′*-dicyclohexylcarbodiimide (DCCD) [30]. The root shedding, caused by cell expansion in the abscission zone, was finished within an hour, a speed much faster than conventional abscission phenomena, which usually requires days. Since the cycloheximide treatment did not inhibit the root abscission induced by NaN_3_, they suggested that rapid root abscission does not need new protein synthesis, offering a novel type of plant abscission phenomenon [30].

Taking progress in NO studies into account, we speculated that NO production in cells treated with NO_2_^−^, NaN_3_, and DNP might have a role in the rapid abscission phenomenon. Thus, we reinvestigated their observations in terms of NO and RNS. In fact, *A. pinnata* was found to emit NO in the presence of NaN_3_ and NO_2_^−^ [21]. Nitrite is now appreciated as an endogenous NO substrate for plants [31] as well as animals [32,33]. Moreover, H_2_O_2_ was found to exhibit a bimodal effect on rapid root abscission [34]. We have proposed a model involving an interplay between RNS and ROS in initiating free radical attack of polysaccharides in the apoplast to account for the rapid root abscission mechanism in *Azolla* plants [35]. Here, we reported the effects of novel H_2_S donors and polysulfides in induction of rapid root abscission that suggests RSS also have a role in the abscission mechanism.

## 2. Materials and Methods

Laboratory cultures of *Azolla pinnata* R, Br. were established from plants collected in April 2018 from a paddy field in Ogimi, Okinawa, Japan. The plants were thoroughly washed to remove attached mud and debris. The plants were then treated with a solution of 0.12% sodium hypochlorite and 0.01% Triton X-100 for 30 min followed by repeated washings in a large volume of distilled water and finally transferred into nutrient culture medium [20]. *A. pinnata* was cultured in a two-fifth strength cobalt-supplemented nitrogen-source-free Hoagland’s E-medium [20]. Medium pH was adjusted to 5.8 with potassium hydroxide (KOH). Plants were grown in a plant growth chamber (Type FLI-2000 H, Eyla, Tokyo, Japan) maintained at 27 °C, 16:8 h light:dark photoperiod and 50 μmol m^−2^ s^−1^ (at plant level) provided by fluorescent lamps (Type FL 40 SBR-A, NEC, Tokyo, Japan). For experiments, fronds were randomly selected from the culture stock and de-rooted manually using forceps. Rootless fronds were placed in the culture medium after rinsing in distilled water and transferred to fresh mediums every 7 days.

Abscission assays were carried out using roots of equal age (i.e., from fronds that had been de-rooted at the same time, 7 days prior to preforming the assay). Three to six fronds (with 20–30 roots) were suspended in a beaker containing 20 mL 10 mM Hepes-KOH (pH 7) or the culture medium (pH 5.8). The abscission test was carried out at room temperature (24 °C) under room light. The chemicals to be tested, the H_2_S chemical donors Na_2_S, GYY4137, 5a, 8l, and 8o (Dojindo Laboratories, Kumamoto, Japan) and the polysulfides Na_2_S_2_, Na_2_S_3_, Na_2_S_4_ (Dojindo Laboratories, Kumamoto, Japan) were subsequently supplied as concentrated stock solutions according to the instruction manuals. The total number of dropped roots following addition of the chemicals was recorded. The abscission response was quantified as the ratio of the detached to the initial number of roots.

H_2_S gas was measured with a handheld O_2_/H_2_S monitor (XOS-326, New Cosmos Electric, Osaka, Japan). The H_2_S that was released into the headspace (10 mL) of a beaker was monitored for 30 s. The H_2_S releasing activity of each solution was expressed as ppm/min.

## 3. Results

### 3.1. Effects of H_2_S Donors on the Root Abscission in Azolla

**Figure 1:**
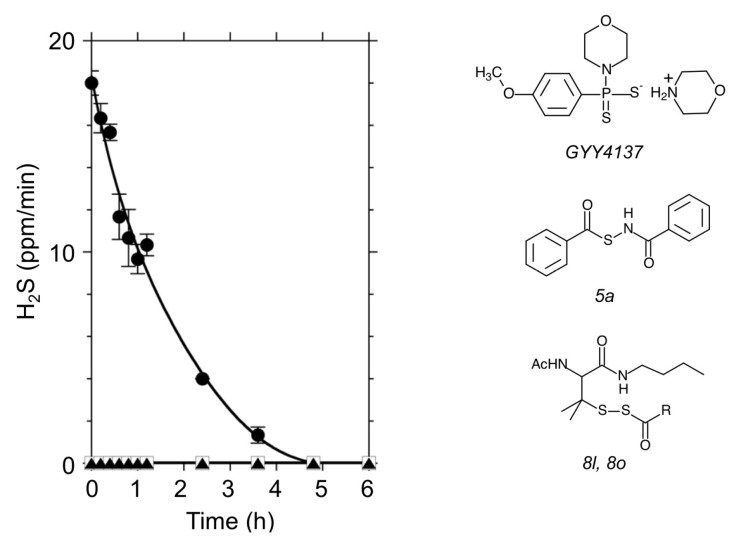
H_2_S gas release from chemical donors. Left panel shows time courses of H_2_S release from a solution containing 10 mM Hepes-KOH (pH 7.0). Abrupt production of H_2_S followed by its decay was observed with Na_2_S (black circle). Under the same conditions, H_2_S released into the air was negligible in GYY4137 (black triangle) and 5a (open square). The H_2_S donor concentration was 100 µM for each. Chemical structures of the H_2_S donors are illustrated on the right.

To investigate biological functions of H_2_S, the application of H_2_S donors would be the first choice for physiological experiments. Sodium hydrosulfide (NaHS) and sodium sulfide (Na_2_S) are inorganic compounds that release H_2_S by hydrolysis. Figure 1 shows a time course of H_2_S emission from a Na_2_S solution. After resolving Na_2_S into a Hepes-KOH buffer at pH 7.0, an abrupt release of H_2_S followed by its decay was observed (Figure 1). This rapid spontaneous reaction makes it difficult to obtain a stable H_2_S concentration for physiological purposes. To overcome this difficulty in application, the synthetic H_2_S donor GYY4137 has been used for stable delivery of H_2_S to cells in many studies. GYY4137 is a Lawesson’s reagent derivative that releases H_2_S via hydrolysis both in vivo and in vitro [36]. In 2011, Xian and co-workers discovered a series of *N*-(benzoylthio) benzamide derivatives that can be activated by thiols to release H_2_S [37,38]. The H_2_S donors 5a, 8l, and 8o are such new tools for exploring biological function of H_2_S. Under the conditions we used, unlike with Na_2_S, we did not measure detectable H_2_S release (ppm) into the air from either GYY4137 or 5a solutions (Figure 1).

**Figure 2:**
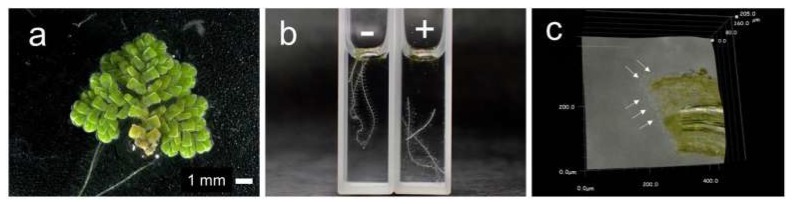
Photographs of root abscission in *Azolla pinnata* induced by the H_2_S donor 5a. (**a**) Photographs of a frond of *A. pinnata*. (**b**) Effect of 5a on the root abscission in 10 mM Hepes-KOH (pH 7.0). Photograph was taken 48 h after the addition of 5a. (−) In the absence of 5a (control), (+) in the presence of 200 µM 5a. (**c**) Expanded cells on the end of a detached root in an abscission zone. The white arrows indicate the expanded cells at end of the detached root.

The water fern *Azolla pinnata* shows a characteristic rapid root abscission phenomenon in response to certain environmental stimuli. In laboratory experiments, a range of chemicals have been reported to be effective in abscising the roots, such as the uncouplers CCCP and DNP, the heme-binding NaN_3_ and KCN [21], the polyamines spermine and spermidine [20], the NO precursor NaNO_2_, and the NO donor spermine NONOate (SNN). Also, H_2_O_2_ appears to be involved in the abscission mechanism [34]. Since the rapid root abscission phenomenon is responsive to both the RNS NO and the ROS H_2_O_2_, it was logical to speculate that H_2_S or RSS might similarly exert an effect. To test this hypothesis, we compared the effects of five H_2_S donors: Na_2_S, GYY4137, 5a, 8l, and 8o on root abscission. Among the compounds tested, the H_2_S donor 5a was found to be effective in detaching the roots (Figure 2). Figure 2b demonstrates the root abscission induced by the H_2_S donor 5a at 200 µM. The detached end of a root abscised by the H_2_S donor 5a showed expanded cells within the abscission zone, which agrees with the morphological features of abscised roots as in previous reports [30,34,35].

**Figure 3:**
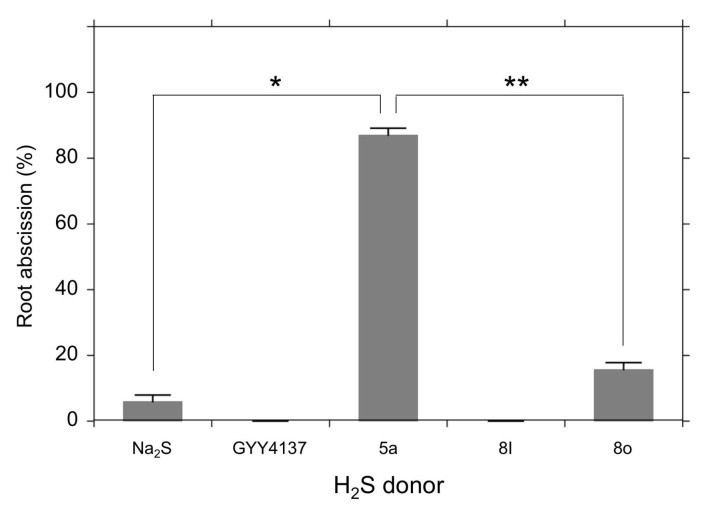
Effects of H_2_S donors on root abscission. Root abscission is represented as % of the detached roots incubated for 24 h with a 10 mM Hepes-KOH (pH 7.0) buffer containing the H_2_S donor (100 µM). The values are means ± SE (*n* = 3). Significant differences are indicated as * or ** (*p* < 0.001).

**Figure 4:**
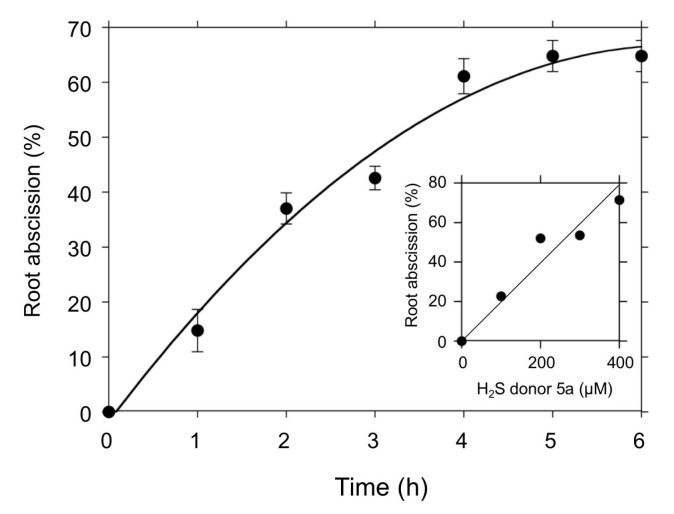
Time course of the root abscission induced by the H_2_S donor 5a. The number of detached roots was counted every hour after the addition of the H_2_S donor 5a (100 µM) into a 10 mM Hepes-KOH (pH 7.0) buffer. Means ± SE (*n* = 3). Inset shows the 5a concentration dependence of the root abscission. The values are expressed as % of the detached roots after one-hour incubation.

Figure 3 compares the effects of the H_2_S donors on the root abscission. With the same concentration (100 µM) and the same incubation time (24 h), the H_2_S donor 5a showed significantly higher root abscission among the five compounds. The effect of GYY4137 was negligible under the conditions used. The abscission induced by 5a was time dependent (Figure 4). With 100 µM 5a, the root abscission continued until 6 h and reached a plateau (Figure 4). Up to 400 µM, the effect of 5a showed concentration dependency (Figure 4, inset). Figure 3 and Figure 4 clearly demonstrate that the H_2_S donor 5a is a novel chemical compound that can induce the root abscission of *Azolla*.

### 3.2. Effects of Polysulfides on the Root Abscission

The H_2_S donors tested in this study released H_2_S by different mechanisms. Both Na_2_S and GYY4137 produce H_2_S by a spontaneous hydrolysis reaction. Although the final amount of H_2_S release may depend on pH, those types of H_2_S donors do not require specific conditions other than the presence of H_2_O. In contrast, the new types of H_2_S donors 5a, 8l, and 8o require the presence of thiols such as cysteine or reduced form of glutathione (GSH) [37]. In fact, no H_2_S emission from 5a in buffered solution was measured, as shown in Figure 1. The mechanisms for H_2_S release from 5a, 8l, and 8o are considered to be: 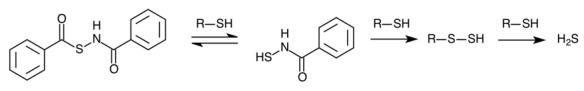
(1)

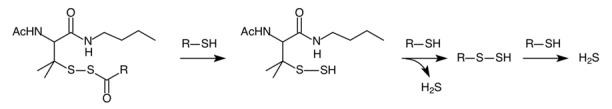
(2)

It is important to note that the H_2_S donor 5a (Equation (1)) as well as 8l and 8o (Equation (2)) decomposes with multiple steps. For each reaction, the presence of thiol (–SH) is required [37]. The difference in structure between 8l and 8o is the R moiety (8l: R = –CH_3,_ 8o: R = –C(CH_3_)_3_). If the compound 5a, in fact, induced root abscission by its chemical reaction, the question arose as to whether the effect relied on H_2_S or on interactions with thiols within the cells. The minimal effect of the spontaneous H_2_S donor Na_2_S and GYY4137 on root abscission led us to check the actions of polysulfides.

**Figure 5:**
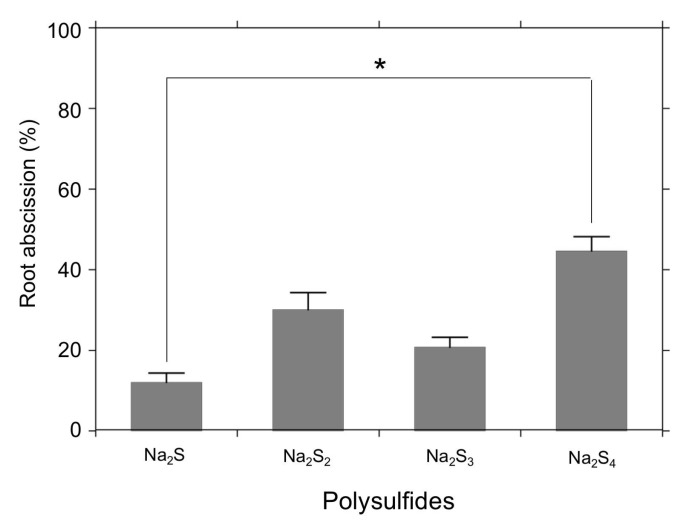
Effects of polysulfides (Na_2_S_n_) on root abscission. Root abscission is represented as % of the total number of the roots. *Azolla* was incubated for 24 h with a 10 mM Hepes-KOH (pH 7.0) buffer containing 500 µM polysulfide (Na_2_S_n_). The values are means ± SE (*n* = 3). A significant difference is indicated as * (*p* < 0.05).

Figure 5 shows effects of sodium polysulfides (Na_2_S_n_) on the root abscission. We compared sodium sulfide (Na_2_S), sodium disulfide (Na_2_S_2_), sodium trisulfide (Na_2_S_3_), and sodium tetrasulfide (Na_2_S_4_). All of these tested compounds showed some abscission-inducing effects, with the polysulfide Na_2_S_4_ being much more effective than Na_2_S in detaching the roots (Figure 5).

### 3.3. d-Cysteine-Induced Root Abscission

**Figure 6:**
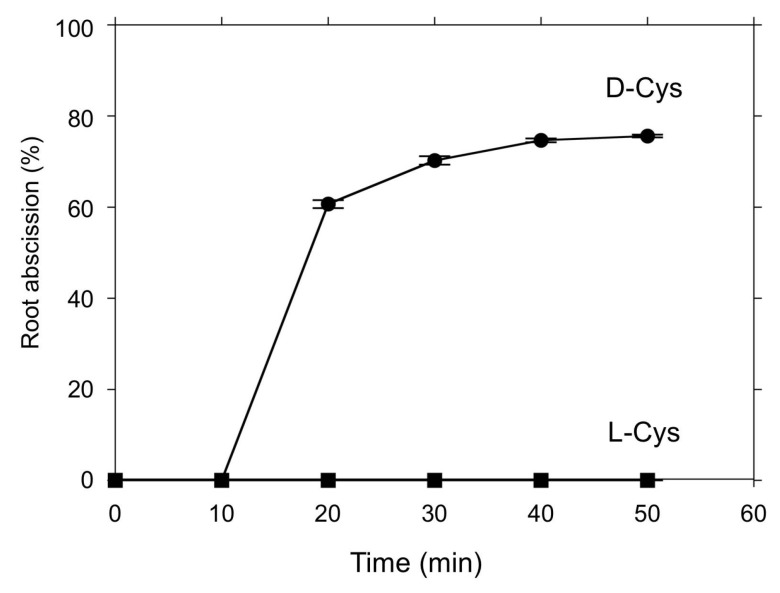
Time course of the d-cysteine-induced root abscission in *A. pinnata*. 3 mM of either d-cysteine (d-Cys) or l-cysteine (l-Cys) was added into the culture medium (pH 5.8). Means ± SE (*n* = 5).

**Figure 7:**
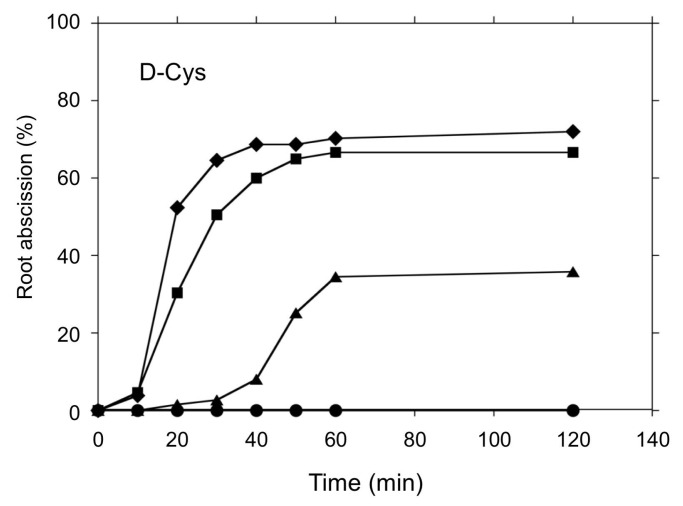
Concentration dependence of d-cysteine on root abscission. d-cysteine at 0.65, 1.25, 2.5, and 5 mM (from the bottom to top) was added into the culture medium (pH 5.8). Means ± SE (*n* = 5).

Although root abscission was induced by the H_2_S donor 5a (Figure 2 and Figure 3) and polysulfides (Figure 4), the abscission proceeded over the course of hours, much slower than for other previously reported chemical inducers [20,30,34]. There are increasing numbers of reports suggesting that l/d-cysteine could be an endogenous substrate for H_2_S synthesis in redox signaling mechanisms [39]. Thus, we tested the effects of l- and d-cysteine on root abscission in *Azolla*. Interestingly, d-cysteine, but not l-cysteine, was found to be efficient in inducing rapid root abscission (Figure 6). Unlike the case of the H_2_S donor 5a or polysulfides, the abscission finished less in than an hour, which is comparable to the effects of other chemical inducers. The effect of d-cysteine showed concentration dependence (Figure 7). The initial speed of the abscission increased with the d-cysteine concentration.

## 4. Discussion

### 4.1. Application of Chemical H_2_S Donors for Inducing Root Abscission

To explore biological functions of H_2_S, physiological experiments with H_2_S chemical donors have been conducted both in plants [13] and mammals [40]. Since Na_2_S and NaHS are relatively cheap, these inorganic H_2_S donors can be applied for large-scale experiments using whole plants [18]. As shown in Figure 1, however, the H_2_S releasing activity of Na_2_S quickly decays such that it is virtually impossible to maintain a stable concentration within a physiological range. Moreover, the inorganic H_2_S donors are extremely moisture sensitive and they are easily oxidized in the presence of O_2_. To compensate for these technical difficulties, many synthetic new H_2_S donors have been developed. In plant science, GYY4137 has been employed to demonstrate physiological functions of H_2_S [13]. We expected that GYY4137 should induce root abscission, but found it does not have a significant abscission inducing activity even at mM concentrations (data not shown). The root abscission activity of Na_2_S was also weak. The novel H_2_S donors 5a, 8l, and 8o need cysteine or GSH for releasing H_2_S into an aqueous phase [38]. Because of this nature, we had not expected the abscission inducing activity of such thiol-activated type of H_2_S donors. However, the H_2_S donor 5a showed reasonably good abscission activity at sub mM concentrations (Figure 4).

Although recent studies have highlighted the “positive” regulatory functions of H_2_S, the molecule is yet cytotoxic and potentially inactivates metalloenzymes. The application of high concentrations of H_2_S from outside of the plants may disturb many enzymatic reactions or metabolisms that are required for the initiation of specific physiological events. We speculate that the structure of 5a bearing aromatic rings facilitates an efficient delivery of the compound to the target due to its lipophilic nature. The requirement of thiols (cysteine or GSH) could further localize the H_2_S production by the compound, thereby minimizing the negative impact of H_2_S. The difference in the effect between 8o and 8l could be also explained by difference in the polarity of the R moiety (Figure 1).

### 4.2. Effects of Polysulfides (Na_2_S_n_) on the Root Abscission

Recent progress in mammalian H_2_S studies has strongly suggested that potential chemical entities regulating biological functions of RSS are polysulfur species (H_2_S_n_), rather than H_2_S itself [40]. A growing number of reports have supported the participation of polysulfidation of cysteine thiols [41,42,43]. To test the involvement of polysulfidation, in this study we applied sodium polysulfides (Na_2_S_n_) for the *Azolla* root abscission experiments. As shown in Figure 4, all polysulfides induced root abscission. The results may imply that polysulfidation of thiol(s) could be involved in the root abscission mechanism. In a good agreement with this aspect, we observed that *S*-methyl methane thiosulfonate (MMTS), which covalently sulfenylates the thiol of cysteine residues, inhibited nitrite-induced root abscission [34]. Moreover, excessive GSH exogenously added was reported to be suppressive to the nitrite-induced root abscission in *A. pinnata* [34]. It should be noted that the polysulfide effects required relatively higher concentrations and longer incubation time compared with the H_2_S donor 5a (Figure 3 and Figure 5). Since H_2_S emission (ppm) into the air was observed even in Na_2_S_2_, Na_2_S_3_, and Na_2_S_4_ solutions (data not shown), we suspect that effects of H_2_S might overlay the actions of the sodium polysulfides. Obviously, further confirmation is necessary to conclude the effects of Na_2_S_n_ have physiological relevance. Like with the H_2_S donor 5a, we may need to wait for new tools of synthetic polysulfides that can effectively mediate a local polysulfidation without spontaneous H_2_S production.

### 4.3. d-Cysteine is a Novel Inducer of Rapid Root Abscsision in Azolla

The present study has demonstrated that d-cysteine is a good inducer of rapid root abscission in *A. pinnata* (Figure 6 and Figure 7), a novel finding that provides an important clue to reveal the root abscission mechanism. d-amino acids had long been thought as a laboratory artifact. Only recently, their biological functions have come to be recognized [39]. In mammals, d-cysteine has been found to be the substrate for H_2_S synthesis catalyzed by 3-mercaptopyruvate sulfurtransferase (3MST) along with d-amino acid oxidase (DAO), namely, the 3MST/DAO pathway [44]. In plants, d-cysteine desulfhydrase (d-CDes) has been suggested to produce H_2_S [45]. The recently sequenced *Azolla filiculoides* genome encodes for a single d-CDes homolog [46]. d-cysteine may induce root abscission via a localized production of H_2_S that may react with an oxidized protein cysteine to form a hydropersulfide [35], or potentially by serving as a substrate for direct enzymatic formation of a persulfide on a cysteine thiol [40]. Interestingly, l-cysteine showed no abscission inducing activity (Figure 6). In *Arabidopsis* plants l-cysteine desulfhydrase activity has been shown to produce H_2_S in the plant cells [47]. It is of a great interest to speculate why *Azolla* does not respond to l-cysteine: is it either due to evolutional [48] or symbiotic [49] reasons?

## 5. Conclusions

Mammalian cells are surrounded by abundant hemeproteins such as blood hemoglobin and muscle myoglobin. Those proteins could maintain low local NO or H_2_S concentrations in the tissues, which is a necessary condition for NO or H_2_S acting as a signaling molecule [1]. In contrast, O_2_-evolving photosynthetic organisms, such as plants, produce H_2_O_2_, NO and H_2_S as the byproducts of the assimilation metabolisms under the light, particularly in stress conditions [39,50]. The lower hemeprotein content in plant tissue enables NO and H_2_S diffuse both in and out of the tissue, thereby permitting sensing of environmental conditions directly through those gases. Presumably, the ROS, RNS and RSS signals would be integrated at the functional thiol groups that are potentially modulated by those reactive molecular species in different forms [40]. In fact, the interplay among ROS, RNS, and RSS produce a variety of reactive products derived from thiols, such as nitrosothiol (SNO), thionitrous acid (HSNO), nitroxyl (HNO), nitropersulfide (SSNO^−^), and polysulfides H_2_S_n_ [40]. This study has, for the first time, demonstrated that d-cysteine is a strong inducer of rapid root abscission in *A. pinnata*. To reveal the molecular mechanism for the d-cysteine-induced root abscission, controlling the chemical redox reactions while monitoring those key molecules will be essential. As we experienced in NO studies [51], physiological experiments with gaseous H_2_S are difficult to handle, and it is sometimes hard to obtain good reproducibility. We suggest that *Azolla* is a good model system to explore RSS-mediated signaling mechanisms in plants because of its tiny size (advantageous for culture [29]), water floating nature (advantageous for pharmacological experiments [20,21]), and rapid response comparable to stomatal responses (advantageous for analysis [35]).

## References

[1] Yamasaki H. (2005). The NO world for plants: Achieving balance in an open system. Plant Cell Environ..

[2] Sakihama Y., Murakami S., Yamasaki H. (2003). Involvement of nitric oxide in the mechanism for stomatal opening in *Vicia faba* leaves. Biol. Plant..

[3] Antoniou C., Savvides A., Christou A., Fotopoulos V. (2016). Unravelling chemical priming machinery in plants: The role of reactive oxygen–nitrogen–sulfur species in abiotic stress tolerance enhancement. Curr. Opin. Plant Biol..

[4] Zhang X., Zhang L., Dong F., Gao J., Galbraith D.W., Song C.-P. (2001). Hydrogen peroxide is involved in abscisic acid-induced stomatal closure in *Vicia faba*. Plant Physiol..

[5] Quan L.J., Zhang B., Shi W.W., Li H.Y. (2008). Hydrogen peroxide in plants: A versatile molecule of the reactive oxygen species network. J. Integr. Plant Biol..

[6] Neill S.J., Desikan R., Clarke A., Hurst R.D., Hancock J.T. (2002). Hydrogen peroxide and nitric oxide as signalling molecules in plants. J. Exp. Bot..

[7] Desikan R., Griffiths R., Hancock J., Neill S. (2002). A new role for an old enzyme: Nitrate reductase-mediated nitric oxide generation is required for abscisic acid-induced stomatal closure in *Arabidopsis thaliana*. Proc. Natl. Acad. Sci. USA.

[8] Desikan R., Cheung M.K., Bright J., Henson D., Hancock J.T., Neill S.J. (2004). ABA, hydrogen peroxide and nitric oxide signalling in stomatal guard cells. J. Exp. Bot..

[9] Ozgur R., Uzilday B., Iwata Y., Koizumi N., Turkan I. (2018). Interplay between the unfolded protein response and reactive oxygen species: A dynamic duo. J. Exp. Bot..

[10] Giles G.I., Tasker K.M., Jacob C. (2001). Hypothesis: The role of reactive sulfur species in oxidative stress. Free Radic. Biol. Med..

[11] Gruhlke M.C.H., Slusarenko A.J. (2012). The biology of reactive sulfur species (RSS). Plant Physiol. Biochem..

[12] Li L., Rose P., Moore P.K. (2011). Hydrogen sulfide and cell signaling. Annu. Rev. Pharmacol. Toxicol..

[13] Lisjak M., Srivastava N., Teklic T., Civale L., Lewandowski K., Wilson I., Wood M.E., Whiteman M., Hancock J.T. (2010). A novel hydrogen sulfide donor causes stomatal opening and reduces nitric oxide accumulation. Plant Physiol. Biochem..

[14] Lisjak M., Teklic T., Wilson I.D., Whiteman M., Hancock J.T. (2013). Hydrogen sulfide: Environmental factor or signalling molecule?. Plant Cell Environ..

[15] De Kok L., Bosma W., Maas F., Kuiper P. (1985). The effect of short-term H_2_S fumigation on water-soluble sulphydryl and glutathione levels in spinach. Plant Cell Environ..

[16] Zhang H., Ye Y.-K., Wang S.-H., Luo J.-P., Tang J., Ma D.-F. (2009). Hydrogen sulfide counteracts chlorophyll loss in sweetpotato seedling leaves and alleviates oxidative damage against osmotic stress. Plant Growth Regul..

[17] Min Y., Qin B.-P., Ping W., Li M.-L., Chen L.-L., Chen L.-T., Sun A.-Q., Wang Z.-L., Yin Y.-P. (2016). Foliar application of sodium hydrosulfide (NaHS), a hydrogen sulfide (H_2_S) donor, can protect seedlings against heat stress in wheat (*Triticum aestivum* L.). J. Integr. Agric..

[18] Kaya C., Ashraf M., Akram N.A. (2018). Hydrogen sulfide regulates the levels of key metabolites and antioxidant defense system to counteract oxidative stress in pepper (*Capsicum annuum* L.) plants exposed to high zinc regime. Environ. Sci. Pollut. Res..

[19] Cohen M.F., Sakihama Y., Takagi Y.C., Ichiba T., Yamasaki H. (2002). Synergistic effect of deoxyanthocyanins from symbiotic fern *Azolla* spp. on *hrmA* gene induction in the cyanobacterium *Nostoc punctiforme*. Mol. Plant Microbe Interact..

[20] Gurung S., Cohen M.F., Fukuto J., Yamasaki H. (2012). Polyamine-induced rapid root abscission in *Azolla pinnata*. J. Amino Acids.

[21] Gurung S., Cohen M.F., Yamasaki H. (2014). Azide-dependent nitric oxide emission from the water fern *Azolla pinnata*. Russ. J. Plant Physiol..

[22] Lumpkin T.A., Plucknett D.L. (1980). Azolla: Botany, physiology, and use as a green manure. Econ. Bot..

[23] Leavitt R.G. (1902). The root-hairs, cap, and sheath of *Azolla*. Bot. Gaz..

[24] Gunning B., Hardham A., Hughes J. (1978). Evidence for initiation of microtubules in discrete regions of the cell cortex in *Azolla* root-tip cells, and an hypothesis on the development of cortical arrays of microtubules. Planta.

[25] Gunning B., Hughes J., Hardham A. (1978). Formative and proliferative cell divisions, cell differentiation, and developmental changes in the meristem of *Azolla* roots. Planta.

[26] Sela M., Tel-Or E., Fritz E., Huttermann A. (1988). Localization and toxic effects of cadmium, copper, and uranium in *Azolla*. Plant Physiol..

[27] Cohen M.F., Yamasaki H., Mazzola M. (2004). Bioremediation of soils by plant-microbe systems. Int. J. Green Energy.

[28] Rao H. (1935). The structure and life-history of *Azolla pinnata* R. Brown with remarks on the fossil history of the hydropterideæ. Proc. Plant Sci..

[29] Kitoh S., Shiomi N., Uheda E. (1993). The growth and nitrogen fixation of *Azolla filiculoides* Lam. in polluted water. Aquat Bot..

[30] Uheda E., Kitoh S. (1994). Rapid shedding of roots from *Azolla filiculoides* plants in response to inhibitors of respiration. Plant Cell Physiol..

[31] Yamasaki H., Sakihama Y., Takahashi S. (1999). An alternative pathway for nitric oxide production in plants: New features of an old enzyme. Trends Plant Sci..

[32] Lundberg J.O., Weitzberg E., Gladwin M.T. (2008). The nitrate–nitrite–nitric oxide pathway in physiology and therapeutics. Nat. Rev. Drug Discov..

[33] Yamasaki H., Watanabe N.S., Fukuto J., Cohen M.F., Tsukahara H., Kaneko K. (2014). Nitrite-dependent nitric oxide production pathway: Diversity of NO production systems. Studies on Pediatric Disorders, Oxidative Stress in Applied Basic Research and Clinical Practice.

[34] Cohen M.F., Gurung S., Birarda G., Holman H.Y.N., Yamasaki H. (2015). Bimodal effect of hydrogen peroxide and oxidative events in nitrite-induced rapid root abscission by the water fern *Azolla pinnata*. Front. Plant Sci..

[35] Cohen M.F., Gurung S., Fukuto J.M., Yamasaki H. (2014). Controlled free radical attack in the apoplast: A hypothesis for roles of O, N and S species in regulatory and polysaccharide cleavage events during rapid abscission by *Azolla*. Plant Sci..

[36] Li L., Whiteman M., Guan Y.Y., Neo K.L., Cheng Y., Lee S.W., Zhao Y., Baskar R., Tan C.-H., Moore P.K. (2008). Characterization of a novel, water-soluble hydrogen sulfide-releasing molecule (GYY4137). Circulation.

[37] Zhao Y., Bhushan S., Yang C., Otsuka H., Stein J.D., Pacheco A., Peng B., Devarie-Baez N.O., Aguilar H.C., Lefer D.J. (2013). Controllable hydrogen sulfide donors and their activity against myocardial ischemia-reperfusion injury. ACS Chem. Biol..

[38] Zhao Y., Wang H., Xian M. (2010). Cysteine-activated hydrogen sulfide (H_2_S) donors. J. Am. Chem. Soc..

[39] Yamasaki H., Cohen M.F. (2016). Biological consilience of hydrogen sulfide and nitric oxide in plants: Gases of primordial earth linking plant, microbial and animal physiologies. Nitric Oxide.

[40] Kimura H. (2019). Signaling by hydrogen sulfide (H_2_S) and polysulfides (H_2_S_n_) in the central nervous system. Neurochem. Int..

[41] Takata T., Tsukuda A., Tsuchiya Y., Akaike T., Watanabe Y. (2019). The active-site cysteine residue of Ca^2+^/calmodulin-dependent protein kinase I is protected from irreversible modification via generation of polysulfidation. Nitric Oxide.

[42] Ihara H., Kasamatsu S., Kitamura A., Nishimura A., Tsutsuki H., Ida T., Ishizaki K., Toyama T., Yoshida E., Hamid H.A. (2017). Exposure to electrophiles impairs reactive persulfide-dependent redox signaling in neuronal cells. Chem. Res. Toxicol..

[43] Akaike T., Ida T., Wei F.-Y., Nishida M., Kumagai Y., Alam M.M., Ihara H., Sawa T., Matsunaga T., Kasamatsu S. (2017). Cysteinyl-tRNA synthetase governs cysteine polysulfidation and mitochondrial bioenergetics. Nat. Commun..

[44] Shibuya N., Koike S., Tanaka M., Ishigami-Yuasa M., Kimura Y., Ogasawara Y., Fukui K., Nagahara N., Kimura H. (2013). A novel pathway for the production of hydrogen sulfide from D-cysteine in mammalian cells. Nat. Commun..

[45] Papenbrock J., Riemenschneider A., Kamp A., Schulz-Vogt H.N., Schmidt A. (2007). Characterization of cysteine-degrading and H_2_S-releasing enzymes of higher plants-from the field to the test tube and back. Plant Biol..

[46] *Azolla filiculoides* Genome Encoding Azfi_s0002.g001593. https://www.fernbase.org/jbrowse_fernbase/?data=data%2Fjson%2FAzolla_asm_v1.1&loc=Azfi_s0002%3A9528035..9533485&tracks=DNA%2CGene%20models-high%20confidence&highlight=.

[47] Álvarez C., García I., Moreno I., Pérez-Pérez M.E., Crespo J.L., Romero L.C., Gotor C. (2012). Cysteine-generated sulfide in the cytosol negatively regulates autophagy and modulates the transcriptional profile in Arabidopsis. Plant Cell.

[48] Li F.-W., Brouwer P., Carretero-Paulet L., Cheng S., De Vries J., Delaux P.-M., Eily A., Koppers N., Kuo L.-Y., Li Z. (2018). Fern genomes elucidate land plant evolution and cyanobacterial symbioses. Nat. Plants.

[49] Eily A.N., Pryer K.M., Li F.W. (2019). A first glimpse at genes important to the *Azolla-Nostoc* symbiosis. Symbiosis.

[50] Corpas F.J., Chaki M., Fernandez-Ocana A., Valderrama R., Palma J.M., Carreras A., Begara-Morales J.C., Airaki M., del Río L.A., Barroso J.B. (2008). Metabolism of reactive nitrogen species in pea plants under abiotic stress conditions. Plant Cell Physiol..

[51] Yamasaki H., Watanabe N.S., Sakihama Y., Cohen M.F., Gupta K.J. (2016). An overview of methods in plant nitric oxide (NO) research: Why do we always need to use multiple methods?. Plant Nitric Oxide: Methods and Protocols.

